# The generation game: Toward the generational genetic stability of continuous culture

**DOI:** 10.1016/j.isci.2025.111787

**Published:** 2025-01-30

**Authors:** Andrew Yiakoumetti, Charlotte Green, Mark Reynolds, John Ward, Gill Stephens, Alex Conradie

**Affiliations:** 1 Sustainable Process Technologies Research Group, Faculty of Engineering, University of Nottingham, Nottingham NG7 2RD, UK; 2 Mitsubishi Chemical Methacrylates, Wilton Centre, Wilton, Redcar TS10 4RF, UK; 3 Department of Biochemical Engineering, University College London, Gower Street, London WC1E 6BT, UK

**Keywords:** Chemical engineering, Biotechnology

## Abstract

Fed-batch bioprocesses are typically deployed to convert renewable feedstocks to bio-based products using metabolically engineered microorganisms. However, for low-value chemicals, fed-batch cultures provide insufficient volumetric productivity to yield commercially viable products. The greater overall volumetric productivity of continuous culture holds techno-economic promise, but the genetic instability of engineered strains has prevented commercial deployment. This study demonstrated the continuous bioproduction of citramalate (CMA) for over 1,000 h at a productivity of 0.32 g_CMA_ g_DCW_^−1^ h^−1^. Plasmid segregational stability was ensured via *infA*-complementation, and structural stability was obtained under phosphate limitation in the chemostat. By contrast, glucose limitation promoted structural plasmid instability. Cost-prohibitive inducers were also avoided by using a constitutive promoter for gene expression. Plasmid-borne expression of CMA synthase delivered enhanced productivity compared to a chromosomal integrant strain also developed in this study. This work advances the techno-economic feasibility of sustainable chemicals manufacturing from renewable feedstocks by engineered strains in microbial cell culture.

## Highlights

• Segregational and structural stability in continuous culture
• Continuous bioproduction of citramalate, a precursor to methacrylic acid
• Essential gene *infA*-complementation in continuous fermentation
• Alternative nutrient limitations in continuous fermentation

## Graphical abstract

**Figure:**
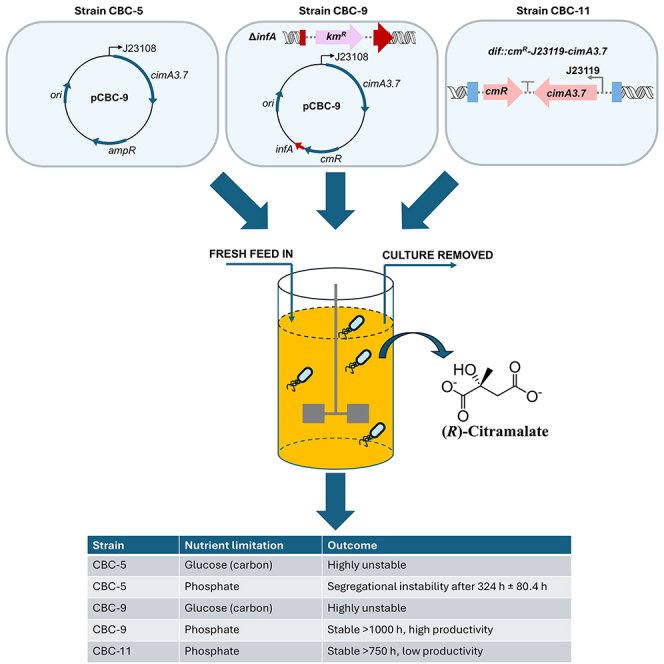

## Introduction

Metabolic engineering allows the bioproduction of industrially useful chemicals from sustainable feedstocks,^1^^,^^2^ providing an alternative to the use of limited fossil resources. Fed-batch bioprocesses are already well established for the production of high value products, such as speciality chemicals and therapeutic proteins, but are less suitable for low-value chemical products because the overall volumetric productivity of fed-batch cultures is too low. The productive period of a fed-batch culture can last up to 75 h, and a further 25 h of turnaround time can be needed to prepare the reactor for the next process cycle. For low-value chemicals, the 25% downtime is sufficient to make the bioprocess unprofitable and prevent commercialization.^3^ Continuous culture could provide a solution by extending the productive period to several hundred hours, thus minimizing the unproductive turnaround time. A limited number of laboratory-scale continuous bioprocesses have been reported for bulk and speciality chemicals production (Table 1), but technical and economic barriers have prevented broader application for manufacturing at scale.

**Table 1:** Previous process development toward establishing continuous culture for small molecule products

Product (Host)	Growth conditions and observations	Reference
L-lysine (*Corynebacterium* sp.)	Complex medium supplemented with glucose; aerobic	Nam-Soon et al.^4^; Ensari et al.^5^
Citric acid (*S. lipolytica*)	Complex medium supplemented with glucose; aerobic	Klasson et al.^6^
Omega-3 (*Y. lipolytica*)	Complex medium supplemented with glucose; aerobic	Xie et al.^7^
L-carnitine (*E. coli*)	Complex medium supplemented with glycerol; aerobic	Bernal et al.^8^
Ethanol (*E. coli*)	Complex medium; anaerobic. Stable production when glucose was used as carbon source. Declining ethanolgenicity observed with mannose, xylose, or a xylose/glucose mixture	Dumsday et al.^9^
Lactic acid (*L. delbrueckii*)	Grown on molasses in complex growth medium; anaerobic	Gupta et al.^10^
1,3-propanediol (*K. pneumoniae*)	Complex medium supplemented with glycerol; anaerobic	Menzel et al.^11^
Succinic acid (*M. succiniciproducens*)	Complex medium supplemented with glucose; anaerobic	Oh et al.^12^
Ethanol and xylitol (*Kluyveromyces* sp.)	Complex medium with glucose and xylose; anaerobic	Kumar et al.^13^
Isopropanol and n-butanol (*C. beijerinckii*)	Complex medium supplemented with glucose; anaerobic	Survase et al.^14^
2,3-butanediol (*K. oxytoca*)	Growth on molasses in complex growth medium under micro-aerobic conditions.	Afschar et al.^15^
Ethanol (*E. coli*)	Complex growth medium supplemented with C5 and C6 sugars; anaerobic. Plasmid stabilized by plasmid addiction system that complements dysfunctional lactate dehydrogenase and pyruvate-formate lyase, and functions only under anaerobic conditions.	Hespell et al.^16^; Dien et al.^17^
2,3-butanediol and isopropanol (*C. necator*)	Growth on CO_2_/H_2_ gas feed with phosphate limiting defined growth medium under aerobic conditions.	Bommareddy et al.^18^
L-phenylalanine (*E. coli*)	Phosphate limiting defined growth medium with glucose as carbon feedstock under aerobic conditions.	Park et al.^19^
L-phenylalanine (*E. coli*)	Growth on glucose in phosphate limiting defined growth medium under aerobic conditions. When glucose limitation or sulfate limitation was used instead, unstable product formation was observed.	Förberg et al.^20^
Gluconic acid (*A. pullulans*)	Two-stage bioreactor with glucose excess in seed reactor. Defined growth medium; aerobic.	Anastassiadis et al.^21^
L-tryptophan (*E. coli*)	Defined growth medium under aerobic conditions, unknown nutrient limitation. Two-stage bioreactor.	Nam et al.^22^
Acetone and n-butanol (*C. acetobutylicum*)	Glucose feedstock with phosphate limiting defined growth medium and a two-stage chemostat. Anaerobic conditions.	Bahl et al.^23^
Ethanol (*C. ljungdahlii*)	Growth in defined medium limited by CO mass transfer. Anaerobic conditions.	Mohammadi et al.^24^
1,3-propanediol (*C. butyricum*)	Long term production from glycerol in phosphate limiting defined growth medium under anaerobic conditions. An anaerobic NADH growth couple ensures that the strain cannot grow on glycerol unless 1,3-propanediol product is formed, simultaneously ensuring both structural stability and plasmid segregational stability.	González-Pajuelo et al.^25^
D-lactate (*E. coli*)	Production from cellobiose in defined growth medium under anaerobic conditions. Lactate production in Δ*pflA* knockout required for NADH reoxidation. Plasmid is only required to express gene *bglC* (encoding β-glucosidase) for cellobiose catabolism.	Aso et al.^26^

The most commonly encountered problem with continuous culture is genetic instability of the metabolically engineered production strain, which can either be due to segregational genetic instability,^27^^,^^28^^,^^29^ or structural genetic instability.^30^^,^^31^^,^^32^^,^^33^ Segregational instability applies to plasmids and their loss at cell division, while structural instability includes the formation of point mutations, deletions and insertions in a genetic sequence. Such mutations can occur in either plasmids or the chromosome. Both segregational and structural instability can lead to non-productive cells that benefit from a reduction in the metabolic burden due to expression of the recombinant genes.^34^ This provides the cells with a competitive growth advantage, so that they outcompete the productive cells, and bring productivity to a halt.^28^^,^^35^^,^^36^

Recombinant plasmids typically harbor an antibiotic resistance marker for plasmid selection, and so segregational stability can be ensured by supplementing the relevant antibiotic to laboratory-scale cultures. However, this approach is prohibitively expensive at industrial scale,^37^^,^^38^ and instead, various genetic approaches have been employed to improve the stability of heterologous production strains in continuous cultures.

One solution is to integrate the heterologous genes into the chromosome to ensure that the genes partition into each daughter cell during replication of the chromosome.^36^^,^^39^^,^^40^ However, chromosomal integration is commonly performed as a single copy integration, and this reduction in gene copy number can lead to decreased gene expression and lower product yield compared to expression from multi-copy plasmids.^41^ The alternative to chromosomal integration is the use of plasmid addiction systems, which can include essential gene complementation,^42^^,^^43^^,^^44^ toxin-antitoxin systems,^45^ and addiction systems linked to substrate uptake^46^ and catabolism.^16^^,^^17^^,^^25^^,^^47^ These create a selection pressure for plasmid maintenance while avoiding the use of antibiotics. However, only toxin-antitoxin systems and essential gene complementation are generally applicable strategies for use with a wide range of host cells and growth conditions, and the use of such addiction systems in continuous culture remains rare and unsatisfactory. For example, a plasmid addiction system based on *trp1*-complementation in *S. cerevisiae* shows vulnerability to perceived cross-feeding,^42^ and another based on *ssb*-complementation in *E. coli* has only been tested for 150 h.^44^ Moreover, neither system was tested in the context of chemicals manufacture. Therefore, a systematic comparison of chromosomal integration with the use of a plasmid addiction system with immunity to cross-feeding is needed in extended continuous culture, within the context of continuous chemicals manufacture.

Reliable scale-up and intensification of continuous culture requires the use of defined growth media, as complex media are costly and vulnerable to batch-to-batch variability. There is some evidence that in chemostat cultures, the growth-limiting nutrient can affect the stability of chemostat cultures. Thus, stable phenylalanine production was achieved when phosphate was used as the growth-limiting nutrient, but product formation declined when either glucose or sulfate limitation was used.^20^ Phosphate limitation also permitted continuous production of other products such as 2,3-butanediol and isopropanol for over 400 h in chemostat culture.^18^ By contrast, carbon limited continuous cultures seem to only be suitable under anaerobic conditions (Table 1) for the production of growth-linked fermentation products such as lactate.^26^ This suggests considerable scope to improve both segregational and structural genetic stability by combining both genetic and physiological approaches, but a systematic comparison of product formation in phosphate- and glucose-limited cultures is needed to confirm this.

Finally, chemical inducers are commonly used at laboratory scale to initiate product formation, but they are too expensive for use at industrial scale and present an economic barrier for the commercialization of bioprocesses. This is especially the case for continuous processes, as the chemical inducers would need to be supplemented continuously to maintain product formation.^48^^,^^49^ Constitutive promoters provide a solution to this, as they enable inducer-free gene expression and product formation. However, fine-tuning of recombinant gene expression is needed during metabolic engineering, as high protein expression levels can have a detrimental effect on cell fitness due to a sub-optimal balance between growth and product formation.^50^^,^^51^

The aim of this study was to develop a continuous cultivation strategy for platform chemical production, integrating both genetic and physiological approaches to ensure segregational and structural stability. The initial objective was to achieve constitutive expression of the pathway to a level that achieves high yield and productivity. The next step was to enable the segregational stability of the expression cassette, comparing a broadly applicable plasmid addiction system for plasmid maintenance with chromosomal integration as an alternative. Finally, the impact of glucose and phosphate limitation on genetic stability was compared using minimal growth media.

**Figure 1:**
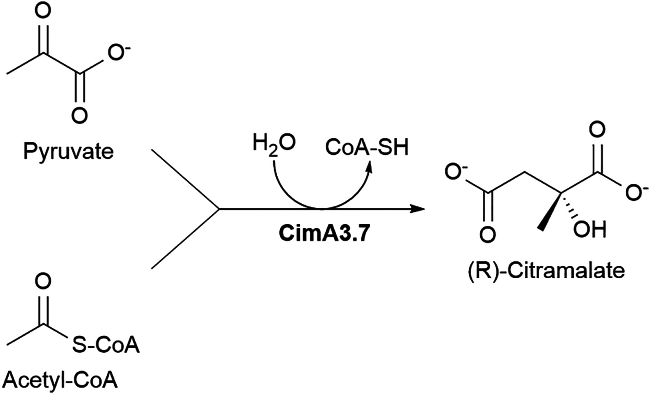
CimA3.7 catalyzed production of the (R)-citramalate anion from the central metabolites, pyruvate, and acetyl-CoA

Citramalate (CMA) was selected as the small molecule case study for three reasons. Firstly, CMA has been produced to high titers (>80 g L^−1^) in fed-batch culture,^52^^,^^53^^,^^54^ providing an industrially relevant carbon flux from central metabolism to a product. Secondly, given that citramalate synthase 3.7 (CimA3.7) converts pyruvate and acetyl-CoA to CMA in a single step (Figure 1), only a single recombinant gene is required in the host strain,^55^ limiting the number of factors that could contribute to genetic instability in continuous culture. Finally, CMA can be used as a platform chemical to produce methacrylic acid (MAA) via decarboxylation in sub-critical water.^56^ Since MAA is widely used to manufacture the ubiquitous polymer, polymethylmethacrylate, which had a European market size valued at $25.5 billion in 2023,^57^ CMA is an industrially relevant product. This study is the first demonstration of a generally applicable plasmid-based system with generational stability for the production of a platform chemical (Figure 1), using fully defined minimal media and without the supplementation of costly chemical inducers.

## Results

### Strain construction and screening

**Figure 2:**
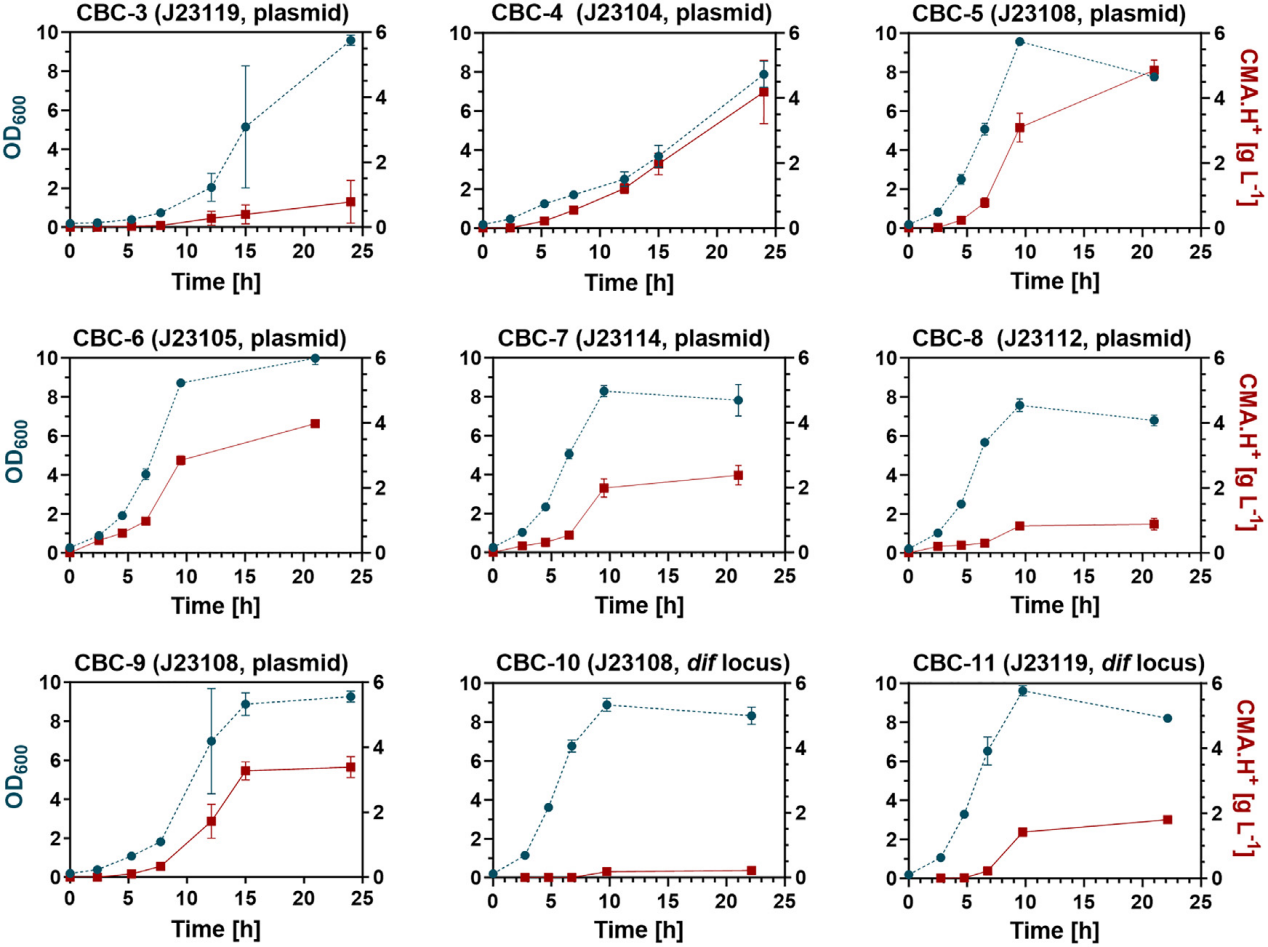
Shake flask screen to identify high titer production strains Engineered strains with varying promoter strengths were grown in Lund medium, in triplicate shake flask cultures. CBC strain numbers (CBC-3–CBC-11) are shown above each graph. The promoter (J23104 to J23119) initiating the expression of *cimA3.7* and the location of the whole expression cassette (plasmid or chromosomal dif locus) are shown alongside the strain number in brackets. OD_600_ measurements are indicated with blue circles joined by dashed lines, and citramalic acid (CMA.H^+^) concentration is indicated by red squares joined by solid lines. CBC-5 demonstrated a favorable balance between productivity and growth, which was maintained after *infA* stabilization, creating CBC-9. CMA concentration and OD values are the mean of biological replicates (*n* = 3) and the error bars show the standard deviation.

Economically feasible continuous cultivation requires the absence of costly chemical inducers. Therefore, a range of promoters was screened to enable the constitutive expression of CMA synthase (encoded by *cimA3.7*). The arabinose inducible Ara_BAD_ promoter in plasmid pBAD24-*cimA3.7*^53^ was replaced with a range of constitutive promoters of varying strengths, viz. J23119, J23104, J23108, J23105, J23114, and J23112 from the Anderson series of promoters, spanning the full range of strengths reported within the iGEM repository for these promoters.^58^ Plasmids pCBC-3 to pCBC-8 (key resources table) each contained one of the aforementioned Anderson promoters in respective order, and were transformed into *E. coli* BW25113 Δ*ldhA*::*kanR* to yield strains CBC-3 to CBC-8 (key resources table). The strains were then screened for CMA production in shake flask culture (Figure 2).

The use of the strongest promoter (J23119) in pCBC-3 resulted in poor cell growth for strain CBC-3 and the lowest final CMA concentration (0.78 ± 0.66 g L^−1^, Figure 2). The use of low strength promoters J23114 in CBC-7 and J23112 in CBC-8 also resulted in low final titers of CMA (2.40 ± 0.30 g L^−1^ and 0.89 ± 0.18 g L^−1^ respectively, Figure 2), despite good growth in shake flasks. Strains CBC-4, CBC-5, and CBC-6 exhibited a favorable balance between productivity and growth. However, CBC-5 (with *cimA3.7* expression under the control of the J23108 promoter) produced the highest final product titer of 4.86 ± 0.31 g L^−1^ after 24 h (Figure 2) and was selected for further study.

In order to improve plasmid segregational stability, strain CBC-5 was modified to enable plasmid addiction via the small essential gene *infA,* which encodes translation initiation factor I.^43^^,^^59^ Plasmid stability has been demonstrated in *infA* stabilized strains for 120 generations in repeated batch cultures and the plasmid addiction system is known to be immune to cross-feeding,^43^ although the addiction system had not been tested in continuous culture. Plasmid pCBC-9 was constructed by encoding *cimA3.7* under the transcriptional control of the J23108 promoter, and *infA* in an operon with a chloramphenicol resistance marker (Figure 5A). Thereafter, strain CBC-9 was constructed by deleting *infA* from the chromosome of cells harboring the pCBC-9 plasmid, such that the plasmid-based *infA* gene complemented the Δ*infA* background and was essential for growth. Strain CBC-9 exhibited a similar growth profile to strain CBC-5, but produced 30% less CMA (3.39 ± 0.32 g L^−1^, Figure 2) compared to strain CBC-5 (4.86 ± 0.31 g L^−1^, Figure 2). It is possible that the replacement of the ampicillin resistance marker with a chloramphenicol resistance marker is responsible for this reduction in titer, since chloramphenicol acetyltransferase may compete with CimA3.7 for acetyl-CoA, even in the absence of chloramphenicol.^60^

Chromosomal integration was also tested as an alternative solution to plasmid segregational instability, by integrating *cimA3.7* into the chromosome. However, the reduced copy number from chromosomal expression, relative to the expression from a multi-copy plasmid, is known to reduce the productivity of bio-processes.^41^^,^^61^ Therefore, as a first iteration design, the strongest promoter from the Anderson series (J23119) was compared with the J23108 promoter of pCBC-5 to increase expression levels. The production cassettes were integrated at the previously validated *dif* locus,^62^^,^^63^^,^^64^ resulting in strains CBC-11 and CBC-10, where CBC-10 expressed *cimA3.7* from the J23108 promoter and CBC-11 expressed *cimA3.7* from the J23119 promoter. In shake flask cultures, strain CBC-10 produced only 0.22 ± 0.05 g L^−1^, while strain CBC-11 produced 1.80 ± 0.13 g L^−1^ CMA, indicating that the stronger promoter was necessary (Figure 2).

### Continuous cultivation of the plasmid-based citramalate production strains

**Figure 3:**
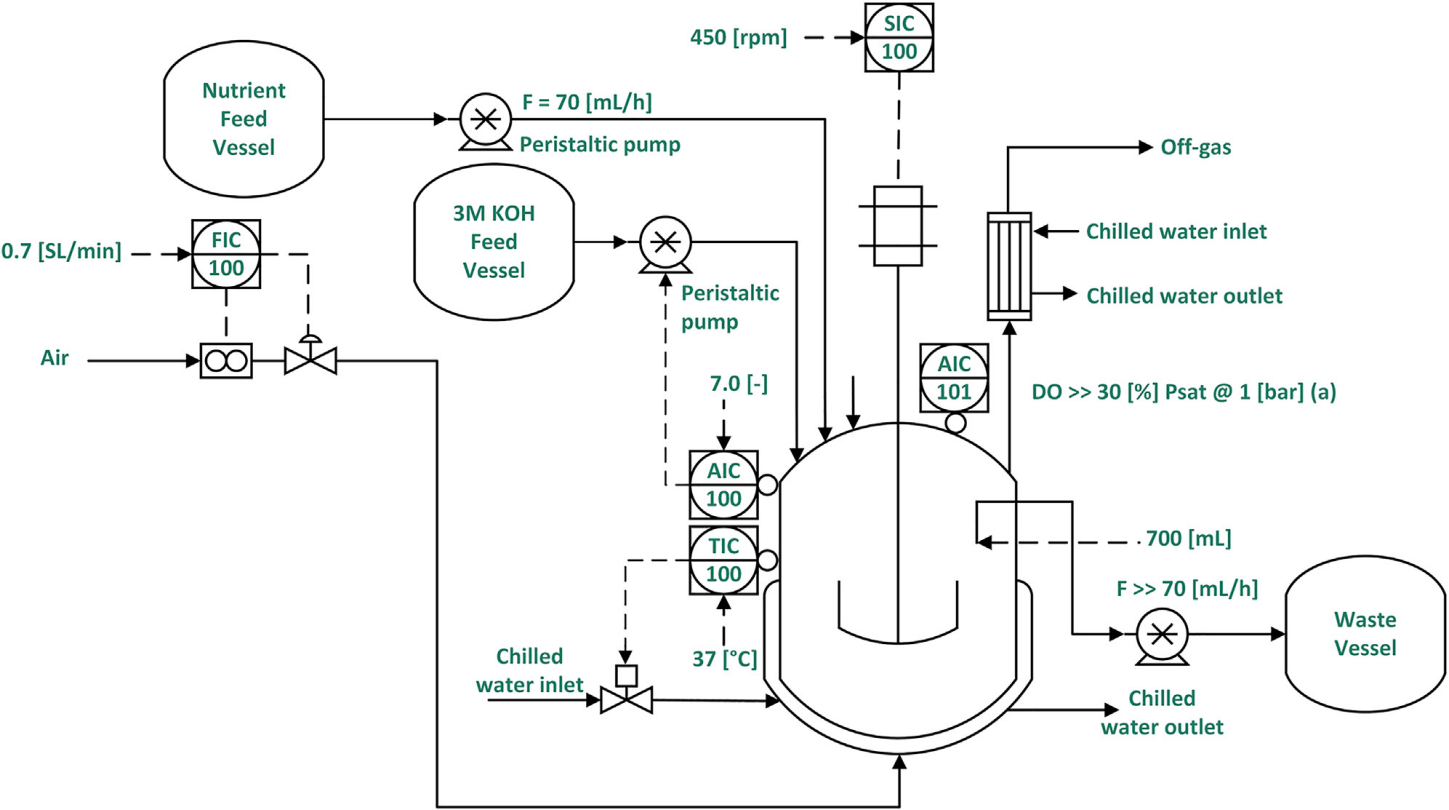
Process flow diagram of the laboratory continuous culture set-up

**Figure 4:**
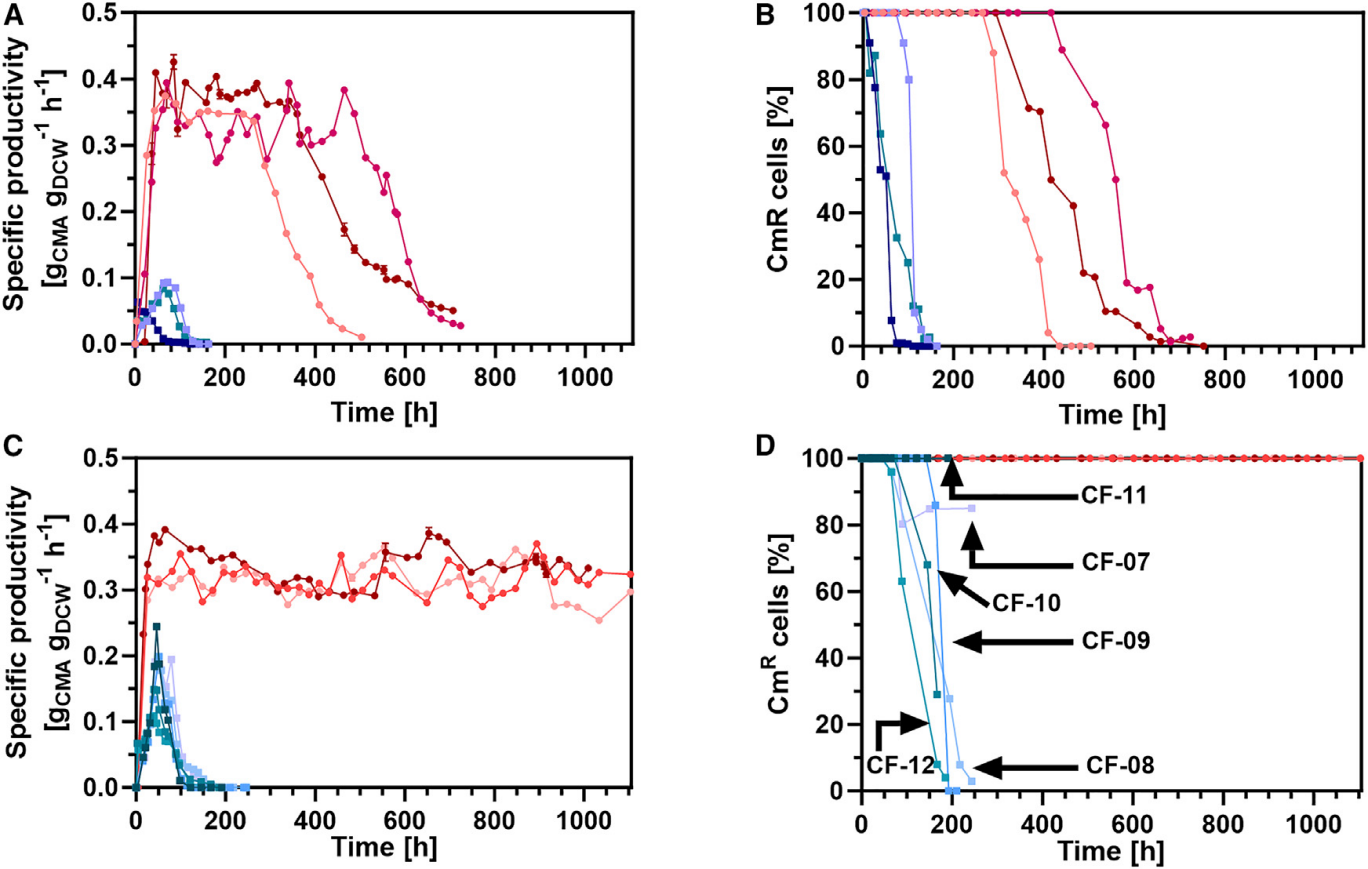
Genetic stability and productivity of CBC-5 and CBC-9 during both glucose limited and phosphate limited continuous culture (A) Specific CMA productivity of CBC-5 under both glucose limitation (blue shades, 3 biological repeats, continuous cultures CC-1–CC-3) and phosphate limitation (red shades, 3 biological repeats, cultures CC-4–CC-6). (B) The percentage of cells with resistance to chloramphenicol in cultures CC-1–CC-3 (blue shades) and in cultures CC-4–CC-6 (red shades). (C) Specific CMA productivity of CBC-9 under both glucose limitation (blue shades, 6 biological repeats, cultures CC-7–CC-12) and phosphate limitation (red shades, 3 biological repeats, cultures CC-13–CC-16). (D) The percentage of cells with resistance to chloramphenicol in cultures CC-7–CC-12 (blue shades) and in cultures CC-13–CC-16 (red shades). Black arrows indicate the glucose limited cultures from which plasmids were isolated and sequenced. Specific productivity values at individual time points for each continuous culture are the mean of values calculated from technical repeats of product concentration (*N* = 3, where *N* is the number of HPLC analysis repeats performed for each sample), using a fixed dilution rate (0.1 h^−1^) and fixed dry cell weight (single measurement, or mean of multiple measurements, as indicated in the supporting data). Error bars for specific productivity represent the standard deviation across the technical repeats.

The productivity and stability of strains CBC-5 (Figure 4A) and CBC-9 (Figure 4C) were assessed in chemostats in the absence of antibiotic selection pressure – as per the experimental setup illustrated in Figure 3—under either glucose limitation or phosphate limitation at a dilution rate of 0.1 h^−1^. Under glucose limitation, the CMA productivity of the non-stabilized strain, CBC-5, reached a maximum at 0.073 ± 0.018 g_CMA_ g_DCW_^−1^ h^−1^ (Figure 4A) but productivity began to decline immediately. An associated loss of plasmid was observed in plasmid retention tests (Figure 4B) and in *Bam*HI digests of plasmids minipreps from samples taken from continuous culture CC-03 (Figure S1A). CMA productivity was lost entirely after 75 h in the first chemostat culture, and after ca. 120 h in the remaining two biological repeats.

CMA productivity and plasmid retention of the non-stabilized strain CBC-5 were improved markedly under phosphate limitation, with an average CMA productivity of 0.35 ± 0.02 g_CMA_ g_DWC_ h^−1^ in the initial steady state period. There was no observed plasmid loss for an average of 324 ± 80.4 h across the three biological repeats (Figure 4B; Table S1). Beyond this cultivation time, productivity began to decline, and cells that had lost the plasmid dominated the culture, noted both by a loss of resistance to chloramphenicol in plasmid retention tests (Figure 4B), and by a decrease in plasmid yield from *Bam*HI digests of plasmid extracts from samples of continuous culture CC-06 (Figure S1B). As CMA productivity ceased, acetate synthesis increased (Figure S2C).

By contrast, cultures of *infA*-stabilized strain CBC-9 remained productive at 0.32 ± 0.01 g_CMA_ g_DCW_^−1^ h^−1^ (∼91% of the steady state productivity of CBC-5) for over 1,000 h (144 generations) under phosphate limitation (Figure 4C; Table S1). All of the bacterial colonies that were screened retained chloramphenicol resistance, suggesting that plasmid was retained throughout the continuous culture (Figure 4D).

Under glucose limitation, the peak productivity of strain CBC-9 with *infA*-complementation was 0.18 ± 0.05 g_CMA_ g_DCW_^−1^ h^−1^, which is a ∼2.5-fold improvement over the peak productivity of the unstabilized strain, CBC-5. However, as with glucose limited cultures of strain CBC-5, the glucose limited continuous cultures of strain CBC-9 were highly unstable. There was a rapid decline in CMA productivity immediately after the peak in productivity was reached, and CMA productivity ceased in all 6 biological replicates after an average of 153 ± 25.4 h (Figure 4C). Furthermore, for 4 out of the 6 biological replicates, chloramphenicol sensitive cells dominated the culture after an average of 202 ± 28.3 h (Figure 4D). Chloramphenicol resistant cells persisted in only two of the glucose limited continuous cultures of CBC-9, continuous cultures CC-07 and CC-11 (Figures 4D and S1C).

The performance of strain CBC-11, in which *cimA*3.7 was integrated into the chromosome and expressed from the J23119 promoter, was also characterized under phosphate limitation (Figure S4). Steady state production of CMA was observed over a period of 776 h (112 generations), when the experiment was terminated. The specific productivity was 0.088 g_CMA_ g_DCW_ h^−1^ (Figure S4A), which is only 27% the productivity of the *infA*-stabilized strain CBC-9 with plasmid-based expression, despite comparable cell densities (Figures S4C and S2A). Acetate was also detected throughout the cultivation of strain CBC-11 (Figure S4B). Given the lower specific productivity and the instabilities observed under glucose limitation for CBC-9 and the much lower productivity of CBC-11 under phosphate limitation, CBC-11 was not characterized under glucose limitation.

### Sequence analysis of infA-stabilized glucose limited continuous cultures

**Figure 5:**
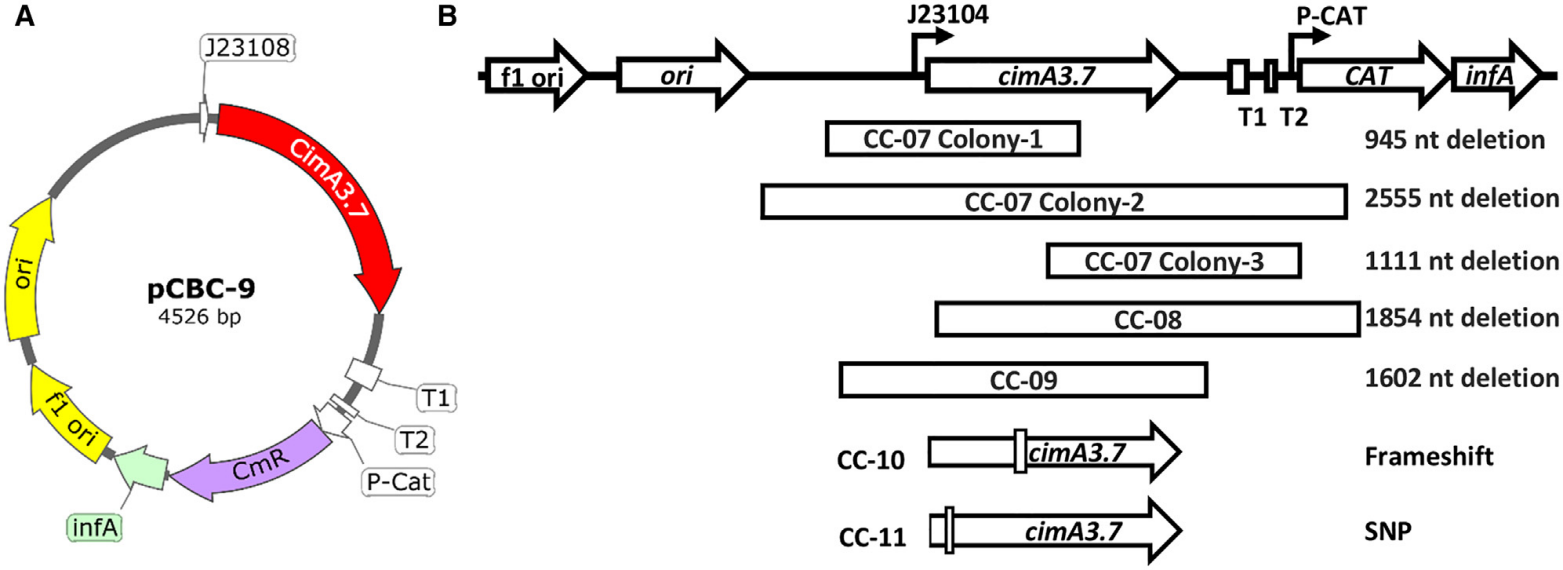
Map of plasmid pCBC-9 and the mutations observed in the plasmid during glucose limited continuous cultures (A) Circular plasmid map for pCBC-9. (B) Cartoon representation of the different mutations observed in pCBC-9 after glucose limited continuous cultures of strain CBC-9. The sequences of isolated mutant plasmids are shown in the supporting data (Mendeley Data: https://doi.org/10.17632/nf3fr6mbgz.1).

Given that cells cannot grow without the plasmid-encoded *infA* expression, plasmid loss could not be the cause of loss of chloramphenicol resistance in glucose-limited continuous cultures of strain CBC-9. Plasmid preparations were therefore made from colonies isolated from different glucose limited cultures of strain CBC-9 and sequenced to determine the causes of loss of chloramphenicol resistance and CMA productivity. The sequence analysis showed a diverse set of structural mutations (illustrated in Figure 5B and summarized in Table S2), where all three of the plasmid isolates from CC-07 (colonies 1–3) were found to have different deletions (Figure 5B; Table S2).

By contrast, the sequences of multiple plasmid isolates from the final broth sample of glucose-limited culture CC-08 yielded the same result (Figure 5B; Table S2), and such population homogeneity was also observed for mutant plasmids from glucose-limited cultures CC-09, CC-10, and CC-11. In continuous cultures CC-07, CC-08, and CC-09, deletions resulted in either the truncation or complete absence of *cimA3.7*. Deletions occurred precisely between regions of contiguous micro-homology spanning 5 to 10 bp, though broader regions of imperfect homology typically surrounded these regions of contiguous micro-homology (Figure S3). In CC-10, a single nucleotide deletion resulted in a frameshift mutation, and in CC-11, a single nucleotide substitution in *cimA3.7* resulted in a missense mutation.

Maintenance of the gene encoding chloramphenicol resistance (*cat*) was variable. The truncation observed in all sequenced colonies of CC-08 included a portion of *cat*, explaining the complete loss of chloramphenicol resistance in CC-08. The truncation in CC-07 colonies 2 and 3 included a portion of *cat*, but the truncation in CC-07 colony 1 did not disrupt *cat*, likely explaining why the loss of chloramphenicol resistance only affected a subset of the population of CC-07. Disruption of *cat* was not observed in CC-09, CC-10, and CC-11 in the sequenced samples, though chloramphenicol resistance was later lost in CC-09 and CC-10 (Figure 4D), suggesting that a subsequent structural mutation had occurred. Interestingly, for the plasmid isolated from colony 2 of CC-07, both the J23108 and CAT promoters were deleted, suggesting that *infA* was sufficiently expressed from an unidentified promoter within the remaining plasmid structure. The entire population of CC-12 became chloramphenicol sensitive, though plasmid isolates were not sequenced from this continuous culture. As expected, sequencing confirmed that plasmid-encoded *infA* was not altered in any of the continuous cultures CC-07 to CC-11.

## Discussion

The commercial feasibility of continuous culture for manufacturing platform chemicals depends upon solving problems with genetic instability. In this study, continuous cultivation and strain development strategies were developed to enable techno-economically feasible productivity and yield alongside prolonged generational stability.

Cost prohibitive chemical inducers were eliminated by developing a constitutive expression system in strain CBC-5 that balanced the split of carbon flux between CMA synthesis and growth. The segregational stability (plasmid retention) of the non-stabilized plasmid in strain CBC-5 was much greater under phosphate limitation compared to glucose limitation, but even so, plasmid loss and a decline in productivity became prominent after 324 ± 80.4 h.

Although antibiotic selection provides a means to select for plasmid-containing cells, the cost of doing so makes this approach unfeasible at scale. Therefore, a plasmid addiction system based on complementation of the small essential gene *infA*^43^^,^^59^ was implemented, yielding strain CBC-9. The *infA*-stabilized plasmid within strain CBC-9 was segregationally stable under both glucose and phosphate limitation. However, under glucose limitation, subpopulations emerged with mis-sense mutations, indel mutations, and more commonly, deletions between regions of micro-homology and caused loss of CMA production. By contrast, under phosphate limitation, the synthesis of CMA by strain CBC-9 was remarkably stable, and the culture remained productive for over 1,000 h (144 generations) with a specific productivity of 0.32 ± 0.01 g_CMA_ g_DCW_^−1^ h^−1^. This study corroborates a prior finding that phosphate limited continuous culture may permit greater structural stability compared to glucose limited continuous culture.^20^

Although segregational stability has been demonstrated in *infA-*stabilized strains for 120 generations in serial transfer batch cultures,^43^ this study is the first demonstration of the efficacy of *infA*-complementation in enabling plasmid retention in true continuous culture. Alternative plasmid addiction systems previously tested in true continuous culture were those based on *trp1*-complementation,^42^ *ssb*-complementation,^44^ or those based on NADH-reoxidation under anaerobic conditions.^16^^,^^17^^,^^25^ The systems based on NADH-reoxidation under anaerobic conditions are host-strain dependent and are not generally applicable to different products, substrates, or growth conditions. By contrast, the system based on *trp1*-complementation is more widely applicable but showed vulnerability to cross feeding, while the system based on *ssb*-complementation was only tested for 150 h. This is an unrealistically short time frame for the technoeconomically viable biomanufacture of chemicals by continuous culture. Therefore, *infA*-complementation appears to be a very promising system to ensure plasmid retention during continuous chemicals manufacture. Future work will explore the general applicability of *infA*-complementation across a broader range of conditions and for the production of a variety of different chemical products.

Chromosomal integration also provides an antibiotic-free alternative to enable segregational stability and did indeed ensure segregational stability and prolonged CMA production under phosphate limitation using strain CBC-11. However, the productivity of CBC-11 was almost four times lower than for strain CBC-9 because of the lower gene dosing, even with the use of the stronger J23119 promoter. Although future work might include the use of stronger promoters^65^^,^^66^ to increase productivity in a chromosomal integrant, stabilization with an addiction system such as *infA*-complementation is arguably simpler, while delivering equally effective genetic stabilization.

In conclusion, a combination of both genetic and physiological strategies was used to develop stable and productive chemostat culture for the production of CMA. Plasmid addiction via *infA*-complementation coupled with phosphate limitation enabled steady state production of CMA for >1,000 h. The plasmid-based system offers the prospect of stable CMA production with <2.4% downtime based on a typical 12–24 h reactor turnaround time. This would have negligible impact upon the volumetric productivity of the bioreactor and, therefore, provides a major improvement in process economics compared to fed-batch culture.

### Limitations of the study

Although this study provides generalizable insights into generational stability in continuous culture, the study’s findings have only been gathered at a dilution rate of 0.1 h^−1^ and at a cultivation temperature of 37°C, and its findings may not be applicable under alternative process conditions. In addition, low cell density cultivation under aerobic cultivation with excess dissolved oxygen and under conditions of uniform mixing (i.e., no spatial concentration gradients) may not be representative of most industrial conditions. Genetically, the strain expressed a single heterologous gene, *cimA3.7*, and many bioprocesses for chemicals production require the expression of multiple genes, which would incur greater metabolic burden on the host. The findings of this study may also not be applicable to other host microorganisms.

## Resource availability

### Lead contact

Requests for further information and resources should be directed to and will be fulfilled by the lead contact, Alex Conradie (a.conradie@ucl.ac.uk).

### Materials availability

• This study did not create any unique reagents.
• Strains and plasmids constructed in this paper can be made available upon request. Requests should be made to the lead contact and corresponding author of this paper, Alex Conradie.

### Data and code availability

• Supporting data including plasmids maps in GenBank format, shake-flask and continuous culture data in a Microsoft Excel file, and plasmid sequence data in FASTA format for chemostat mutated variants of plasmid pCBC-9 (highlighted in Figure 5B), can be accessed on Mendeley Data: https://doi.org/10.17632/nf3fr6mbgz.1.
• This study did not generate original code.
• Any additional information required to reanalyze the data reported in this paper is available from the lead contact upon request.

## Acknowledgments

We would like to thank Professor Stephen Oliver from the University of Cambridge and Professor Eriko Takano from the University of Manchester, who alongside Mitsubishi Chemical UK and Ingenza Ltd, as well as our project manager Andrew Wells, made valuable contributions to this work. This work was made possible by the Industrial Biotechnology (IB) Catalyst grant BB/N023773/1 funded by 10.13039/501100006041Innovate UK, the Biological Sciences Research Council (BBSRC) and the Engineering and Physical Sciences Research Council (EPSRC) (A.Y., C.G., J.W., G.S., and A.C.). The authors gratefully acknowledge support received from the University of Nottingham Research Beacon of Excellence: Green Chemicals.

## Author contributions

Conceptualization, A.Y., C.G., J.W., G.S., and A.C.; data curation, A.Y. and C.G.; formal analysis, A.Y., C.G., and A.C.; funding acquisition, G.S. and A.C.; investigation, A.Y. and C.G.; methodology, A.Y., C.G., G.S., and A.C.; supervision, G.S., J.W., and A.C.; visualization, A.Y., C.G., and A.C.; writing - initial draft, A.Y. and C.G.; writing - review and editing, A.Y., C.G., M.R., J.W., G.S., and A.C.

## Declaration of interests

The authors declare no competing interests.

## STAR★Methods

### Key resources table

**Table:** 

REAGENT or RESOURCE	SOURCE	IDENTIFIER
**Bacterial and virus strains**		

*E. coli* BW25113 Δ*ldhA*::*Km*^R^	KEIO Collection	JW1375
*E. coli* BW25113 Δ*ldhA*::FRT	This paper	N/A
Strain CBC-3: *E. coli* BW25113 Δ*ldhA*::*Km*^R^ pCBC-3	This paper	N/A
Strain CBC-4: *E. coli* BW25113 Δ*ldhA*::*Km*^R^ pCBC-4	This paper	N/A
Strain CBC-5: *E. coli* BW25113 Δ*ldhA*::*Km*^R^ pCBC-5	This paper	N/A
Strain CBC-6: *E. coli* BW25113 Δ*ldhA*::*Km*^R^ pCBC-6	This paper	N/A
Strain CBC-7: *E. coli* BW25113 Δ*ldhA*::*Km*^R^ pCBC-7	This paper	N/A
Strain CBC-8: *E. coli* BW25113 Δ*ldhA*::*Km*^R^ pCBC-8	This paper	N/A
Strain CBC-9: *E. coli* BW25113 Δ*ldhA*::FRT Δ*infA*::*Km*^R^ pCBC-9	This paper	N/A
Strain CBC-10: *E. coli* BW25113 Δ*ldhA*::FRT *dif*::*Cm*^R^-J23108-*cimA*3.7	This paper	N/A
Strain CBC-11: *E. coli* BW25113 Δ*ldhA*::FRT *dif*::*Cm*^R^-J23119-*cimA*3.7	This paper	N/A

**Chemicals, peptides, and recombinant proteins**		

LB broth (Luria Broth base, Miller’s modified)	Sigma	Cat# L1900
LB Agar (LB agar, Miller)	Sigma	Cat# L3027
Agar	Sigma	CAS: 9002-18-0
Ammonium Sulfate	Fisher	CAS: 7783-20-2
Potassium phosphate dibasic (anhydrous)	Fisher	CAS: 7758-11-4
Sodium phosphate monobasic dihydrate	Fisher	CAS: 13472-35-0
Ammonium citrate dibasic	Fisher	CAS: 3012-65-5
Magnesium sulfate heptahydrate	Fisher	CAS: 10034-99-8
D-Glucose	Fisher	CAS: 50-99-7
Ethylenediaminetetraacetic acid disodium salt dihydrate (EDTA disodium salt dihydrate)	Fisher	CAS: 6381-92-6
Calcium chloride dihydrate	Fisher	CAS: 10035-04-8
Iron (III) chloride	Fisher	CAS: 7705-08-0
Zinc sulfate heptahydrate	Fisher	CAS: 7446-20-0
Copper(II) sulfate pentahydrate	Fisher	CAS: 7758-99-8
Manganese(II) sulfate monohydrate	Fisher	CAS: 10034-96-5
Cobalt(II) chloride hexahydrate	Fisher	CAS: 7791-13-1
Hydrochloric acid (HCl)	Fisher	CAS: 7647-01-0
Potassium hydroxide	Fisher	CAS: 1310-58-3
Potassium phosphate monobasic	Fisher	CAS: 7778-77-0
Ammonium chloride	Fisher	CAS: 12125-02-9
Magnesium sulfate heptahydrate	Fisher	CAS: 10034-99-8
Calcium chloride	Fisher	CAS: 10043-52-4
Manganese(II) chloride tetrahydrate	Fisher	CAS: 13446-34-9
Iron(II) sulfate heptahydrate	Fisher	CAS: 7782-63-0
Ammonium molybdate tetrahydrate	Fisher	CAS: 12054-85-2
Tris Base	Fisher	CAS: 77-86-1
Sodium chloride	Fisher	CAS: 7647-14-5
Polypropylene glycol 2000	Fisher	CAS: 25322-69-4
Carbenicillin disodium salt	Melford	CAS: 4800-94-6
Chloramphenicol	Melford	CAS: 56-75-7
Kanamycin monosulfate [Kanamycin A]	Melford	CAS: 25389-94-0
L-arabinose	Sigma	CAS: 5328-37-0

**Critical commercial assays**		

Q5 DNA polymerase	NEB UK Ltd	Cat#: M0491
NEBuilder® HiFi DNA Assembly Master Mix	NEB UK Ltd	Cat#: E2621
FastDigest *Bam*HI (*Bam*HI)	ThermoFisher Scientific	Cat#: FD0054
FastDigest *Nhe*I	ThermoFisher Scientific	Cat#: FD0974
FastDigest *Xho*I	ThermoFisher Scientific	Cat#: FD0694
Antarctic phosphatase	NEB UK Ltd	Cat#: M0289
T4 Polynucleotide kinase	NEB UK Ltd	Cat#: M0201
T4 DNA Ligase	NEB UK Ltd	Cat#: M0202S
GenElute™ Bacterial Genomic DNA Kit	Sigma Aldrich	Cat#: NA2110

**Deposited data**		

Recombinant DNA GenBank maps (plasmids pCBC-2 to pCBC-9, *infA* knockout cassette, *Cm*^R^-J23108-*cimA*3.7 knock-in cassette, *Cm*^R^-J23119-*cimA*3.7 knock-in cassette); shake-flask and continuous culture data; mutated plasmid sequencing in FASTA format.	This paper; Mendeley Data	Mendeley Data: https://doi.org/10.17632/nf3fr6mbgz.1

**Oligonucleotides**		

Oligonucleotides OLIGO-1 to OLIGO-31, see Table S3	This paper	N/A

**Recombinant DNA**		

Plasmid: pBAD24-*cimA3.7*	Webb et al.^53^	N/A
Plasmid: pSTV28	TaKaRa Bioscience	Cat# 3331
Plasmid: pKD32	Datsenko and Wanner^67^	N/A
Plasmid: pKD4	Datsenko and Wanner^67^	ADDGENE: 45605
Plasmid: pKD46	Datsenko and Wanner^67^	N/A
Plasmid: pCP20	Cherepanov and Wackernagel^68^	N/A
Plasmid: pCBC-2	This paper	N/A
Plasmid: pCBC-3	This paper	N/A
Plasmid: pCBC-4	This paper	N/A
Plasmid: pCBC-5	This paper	N/A
Plasmid: pCBC-6	This paper	N/A
Plasmid: pCBC-7	This paper	N/A
Plasmid: pCBC-8	This paper	N/A
Plasmid: pCBC-9	This paper	N/A
Double-stranded linear DNA: *infA* knockout cassette	This paper	N/A
Double-stranded linear DNA: *Cm*^R^-J23108-*cimA*3.7 knock-in cassette	This paper	N/A
Double-stranded linear DNA: *Cm*^R^-J23119-*cimA*3.7 knock-in cassette	This paper	N/A

**Software and algorithms**		

OpenLab ChemStation	Agilent	https://www.agilent.com/en/product/software-informatics/analytical-software-suite/chromatography-data-systems/openlab-chemstation

### Method details

#### Growth media and agar plates

LB broth (Luria Broth base, Miller’s modified) and LB agar (LB agar, Miller) were prepared as per the manufacturer’s instructions. For growth of strains CBC-3 – CBC-8, LB broth and LB agar were supplemented with carbenicillin (50 mg L^−1^).

Lund medium contained (per L): 2 g ammonium sulfate, 14.6 g potassium phosphate dibasic (anhydrous), 3.6 g sodium phosphate monobasic di-hydrate, 0.5 g ammonium citrate dibasic, 0.493 g magnesium sulfate heptahydrate, 10 g D-glucose, and 2 mL 500× Trace Element Solution. The 500× Trace Elements Solution contained (per L): 22.3 g EDTA disodium salt dihydrate, 0.5 g calcium chloride dihydrate, 10.03 g iron(III) chloride, 0.18 g zinc sulfate heptahydrate, 0.16 g copper(II) sulfate pentahydrate, 0.15 g manganese(II) sulfate monohydrate, 0.18 g cobalt(II) chloride hexahydrate. Lund medium was adjusted to pH 7 with hydrochloric acid and filter sterilized. Lund medium was supplemented with carbenicillin (50 mg L^−1^) for cultivation of strains CBC-3 to CBC-8, or with chloramphenicol for strains CBC-9 (34 mg L^−1^), CBC-10 (20 mg L^−1^) and CBC-11 (20 mg L^−1^).

MS broth (1 L, for shake-flask cultures) was composed of 760 mL MS Solution A, 200 mL MS Solution B and 40 mL of 125 g L^−1^ D-glucose. MS Solution A contained (per 760 mL) 2 g potassium phosphate monobasic and 2 mL modified Vishniac trace elements, and was adjusted to pH 7. MS Solution B contained (per 200 mL) 4 g ammonium chloride and 0.4 g magnesium sulfate heptahydrate. MS Solution A, MS Solution B and the glucose solution were autoclaved separately and combined once cool. Modified Vishniac trace elements contained, per L: 63.7 g EDTA disodium salt dihydrate, 2.2 g zinc sulfate, 5.54 g calcium chloride, 5.06 g manganese(II) chloride tetrahydrate, 5 g iron(II) sulfate heptahydrate, 1.1 g ammonium molybdate tetrahydrate, 1.57 g copper(II) sulfate pentahydrate and 1.61 g cobalt chloride hexahydrate. Modified Vishniac trace elements solution was adjusted to pH 6 and stored at 4°C. MS medium for shake-flask cultures was supplemented with carbencillin (50 mg L^−1^) for cultures of strain CBC-5.

Glucose-limiting MS medium for continuous culture was prepared in a similar manner as MS medium for shake-flask cultures but was scaled up to 20 L and contained polypropylene glycol 2000 (PPG2000), and the pH of MS Solution A was also not pH adjusted. MS solution A was autoclaved together with 5 mL PPG2000 in a 20 L borosilicate pot with a magnetic stirrer *in situ*. MS Solution B and the glucose solution were autoclaved separately and transferred to the 20 L pot once cool.

Phosphate-limiting MS medium for continuous culture was similar to glucose-limiting MS medium but contained (per 20 L) only 1.36 g of potassium phosphate monobasic and 21.18 g potassium chloride, to ensure a potassium ion concentration that was equimolar with that of glucose-limiting MS medium.

For patch plating, MS agar (1 L) was prepared as per MS broth (1 L), but 15 g agar was added to the 780 mL MSA prior to autoclave. MS agar was optionally supplemented with carbenicillin (50 mg L^−1^) or chloramphenicol (34 mg L^−1^).

#### Plasmid and strain construction

All strains and plasmids utilized and constructed in this study are described in the key resources table. Details of all oligonucleotides are provided in Table S3. All PCR reactions were performed using Q5 High-Fidelity DNA polymerase. All ligations were performed using T4 DNA ligase. All 5′-phosphorylations of oligonucleotides were performed using T4 polynucleotide kinase using 300 pmol of 5′ termini in 50 μL reactions.

Plasmid pCBC-2 was constructed by first amplifying plasmid pBAD24-cimA3.7 by PCR using oligonucleotides OLIGO-1 and OLIGO-2 as primers. The resulting PCR fragment was digested with *Bam*HI and *Nhe*I, before dephosphorylation with Antarctic Phosphatase to yield the vector backbone for plasmid pCBC-2. A short DNA fragment containing the T7 promoter, an *Xho*I restriction site, and compatible cohesive ends to *Bam*HI and *Nhe*I restriction sites was then constructed by first phosphorylating oligonucleotides OLIGO-3 and OLIGO-4 with T4 polynucleotide kinase and annealing them. The phosphorylated oligonucleotides (10 μM each) were annealed in 1× Oligonucleotide Annealing Buffer in a thermocycler, programmed to hold at 95°C for 3 min, followed by 90°C for 2 min, and to then decrease by 2°C every 2 min until 14°C, before finally holding at 4°C. 10× Oligonucleotide Annealing Buffer was prepared as described previously,^69^ by adding 400 μL of 121.136 g L^−1^ Tris Base solution (titrated to pH 8 with hydrochloric acid), 80 μL of 186.12 g L^−1^ EDTA disodium salt dihydrate solution (titrated to pH 8 with hydrochloric acid) and 800 μL of 146.1 g L^−1^ sodium chloride solution to 2720 μL of nuclease-free water. Each individual solution was autoclaved to destroy nucleases. The resulting short double stranded DNA fragment containing the T7 promoter and a *Xho*I restriction site was then ligated to the vector backbone for plasmid pCBC-2 using T4 DNA ligase, as per the manufacturer’s instructions, to yield plasmid pCBC-2. The sequence of plasmid pCBC-2 was verified by Sanger sequencing.

Plasmids pCBC-3 to pCBC-8 were then constructed by first digesting plasmid pCBC-2 with *Bam*HI and *Xho*I, before dephosphorylating the 4.4 kbp fragment with Antarctic Phosphatase to yield the vector backbone for plasmids pCBC-3 to pCBC-8. Short DNA fragments encoding promoters J23119, J23104, J23108, J23105, J23114, J23112 were constructed by first phosphorylating and annealing corresponding oligonucleotide pairs, in the same way that the T7 promoter fragment was constructed. The corresponding oligonucleotide pairs for the above promoters are listed in Table S3. The promoter fragments for promoters J23119, J23104, J23108, J23105, J23114 and J23119 were ligated into the 4.4 kbp vector backbone derived from the *Bam*HI and *Xho*I digestion of plasmid pCBC-2 to yield plasmids pCBC-3 to pCBC-8, respectively. The promoter and 200 nucleotides of flanking DNA was sequenced by Sanger sequencing for each plasmid.

Plasmid pCBC-9 was constructed by HiFi assembly of 3 DNA fragments using the NEBuilder HiFi DNA Assembly Cloning Kit, according to the manufacturer’s instructions, and using a 60 min incubation time. The first DNA fragment was amplified from plasmid pCBC-5 by PCR using oligonucleotides OLIGO-17 and OLIGO-18 as primers. The second DNA fragment encoded the essential gene *infA* and was amplified from *E. coli* BW25113 genomic DNA using oligonucleotides OLIGO-19 and OLIGO-20 as primers. *E. coli* BW25113 genomic DNA was prepared from an overnight cell culture of *E. coli* BW25113 using the GenElute Bacterial Genomic DNA Kit. Finally, the third DNA fragment encoded a chloramphenicol resistance marker and was amplified from plasmid pSTV28 using oligonucleotides OLIGO-21 and OLIGO-22 as primers. Plasmid pCBC-9 was sequenced by Sanger sequencing.

Strains CBC-3 to CBC-8 were constructed by transforming electrocompetent *E. coli* BW25113 *ΔldhA*::*km*^R^ with plasmids pCBC-3 to pCBC-8 by electroporation, respectively. Transformed cells were plated onto LB agar plates supplemented with carbenicillin (50 mg L^−1^).

Strains CBC-9 to CBC-11 were constructed by Lambda RED recombineering^67^ of strain *E. coli* BW25113 Δ*ldhA::*FRT. Strain *E. coli* BW25113 Δ*ldhA::*FRT was constructed by curing the kanamycin resistance marker from strain *E. coli* BW25113 Δ*ldhA::Km*^R^ cells by Flippase mediated site-specific recombination between FRT-sites. Electrocompetent *E. coli* BW25113 Δ*ldhA::Km*^R^ cells were transformed with pCP20, and outgrowth was performed in SOC medium at 30°C with shaking at 250 rpm for 1 h. Cells were then plated on LB agar plates supplemented with carbenicillin, and incubated overnight at 30°C. LB medium (10 mL) without any antibiotic supplementation was then inoculated from a single colony of *E. coli* BW25113 Δ*ldhA::Km*^R^ and incubated at 42°C with shaking at 250 rpm for 16 h. Culture (100 μL) was then plated onto LB agar plates without supplementation of any antibiotics. This plate was incubated at 37°C for 24 h. Colonies were patched onto LB agar supplemented with kanamycin to confirm the loss of the chromosomal kanamycin resistance marker, as well as onto LB agar supplemented with carbenicillin to confirm the loss of pCP20, and onto LB agar without antibiotics, for clonal maintenance of the successfully cured strains.

To construct strain CBC-9, plasmids pCBC-9 and pKD46 were co-transformed into *E. coli* BW25113 Δ*ldhA*::FRT and outgrowth was performed at 30°C with shaking at 250 rpm for 3 h before plating on LB agar supplemented with glucose (5 g L^−1^), chloramphenicol (34 mg L^−1^) and carbenicillin (100 mg L^−1^). Plates were incubated overnight at 30°C, and a single colony was used to inoculate 10 mL LB supplemented with chloramphenicol (34 mg L^−1^) and carbenicillin (100 mg L^−1^). This 10 mL culture was incubated for 12–16 h at 30°C with shaking at 250 rpm and was then used to inoculate a 100 mL LB culture supplemented with chloramphenicol (34 mg L^−1^), carbenicillin (100 mg L^−1^) and L-arabinose (1.5 g L^−1^) to an OD_600_ of 0.05. This culture was incubated at 30°C until OD_600_ 0.3–0.4. Cells were then harvested by centrifugation (2000 × *g*, 4°C, 10 min) and re-suspended in 100 mL ice-cold sterile water. Cell harvesting was repeated thrice more, re-suspending once in 10 mL ice-cold 10% (v/v) glycerol, then in 1 mL ice-cold 10% (v/v) glycerol, and finally in 100 μL ice-cold 10% (v/v) glycerol. Resuspended cells (40 μL) were then mixed with 500 ng of the *infA*-knockout cassette. This knockout cassette was amplified by PCR from plasmid pKD4 using oligonucleotides OLIGO-23 and OLIGO-24 as primers, as previously described.^70^ The cell-DNA mixture was transferred to separate ice-cold 2 mm gap electroporation cuvettes, and electroporation was performed at 2.5 kV, 200 Ω and 25 μF. Cells were then mixed immediately with pre-warmed SOC medium (1 mL) supplemented with L-arabinose (1.5 g L^−1^), and outgrowth was performed at 30°C with shaking at 250 rpm for 3 h. Cells were then harvested by centrifugation at 5000 × *g* for 10 min, and resuspended in 100 μL SOC medium. The resuspended cells were plated onto LB agar plates supplemented with kanamycin (25 mg L^−1^) and incubated overnight at 37°C.

Strains CBC-10 and CBC-11 were constructed in a similar manner to which strain CBC-9 was constructed, but *E. coli* BW25113 Δ*ldhA::*FRT was only transformed with pKD46 and not pCBC-9. Therefore, no chloramphenicol was added to LB agar or LB broth for the maintenance of plasmid pCBC-9. Instead of using the *infA* knockout cassette, the *Cm*^R^-J23108-*cimA3.7* (for strain CBC-10) and *Cm*^R^-J23108-*cimA3.7* (for strain CBC-11) knock-in cassettes were used. Each knock-in cassette was constructed by overlap extension PCR of two DNA fragments. A DNA fragment containing a chloramphenicol resistance marker (*Cm*^R^-fragment) was common to both knock-in cassettes and was amplified from plasmid pKD32 by PCR using oligonucleotides OLIGO-25 and OLIGO-26 as primers. The J23108-*cimA3.7* fragment for the *Cm*^R^-J23108-*cimA3.7* knock-in cassette was amplified from plasmid pCBC-5 by PCR using oligonucleotides OLIGO-27 and OLIGO-28 as primers. The J23119-*cimA3.7* fragment for the *Cm*^R^-J23119-*cimA3.7* knock-in cassette was amplified from plasmid pCBC-3 by PCR using oligonucleotides OLIGO-29 and OLIGO-28 as primers. The *Cm*^R^-J23108-*cimA3.7* knock-in cassette was constructed by overlap extension PCR of the *Cm*^R^ fragment with the J23108-*cimA3.7* fragment. Similarly, the *Cm*^R^-J23119-*cimA3.7* knock-in cassette was constructed by overlap extension PCR of the *Cm*^R^ fragment with the J23119-*cimA3.7* fragment. In both cases, overlap extension PCR was performed using oligonucleotides OLIGO-30 and OLIGO-31 as external primers. At the final step, the electroporated cells were plated onto LB agar supplemented with chloramphenicol (20 mg L^−1^) rather than carbenicillin to select for the *Cm*^R^-J23108-*cimA3.7* and *Cm*^R^-J23119-*cimA3.7* integrants.

#### Shake-flask screening

For shake flask screening of strains, stock cultures were first streaked onto selective LB agar plates and incubated overnight at 37°C, before single colonies were transferred to 10 mL LB medium in 50 mL Falcon tubes, and grown for 12 h–16 h at 37°C with shaking at 250 rpm. These starter cultures were used to inoculate 50 mL Lund medium in 250 mL baffled shake flasks, to an initial OD_600_ of 0.2. Cultures were incubated at 37°C with shaking at 250 rpm, and sampled for analysis every 2 h–3 h for the first 10 h, and then once more after 10 h–15 h.

#### Continuous culture in chemostats

Continuous (chemostat) cultures were carried out in 1.3 L BioFlo/CelliGen 115 bioreactors (New Brunswick Scientific), equipped with a single Rushton impeller. A diagram of the set-up is shown in Figure 3. Continuous cultures with a working volume of 0.7 L were cultivated at 37°C, with an air flow of 0.7 L min^−1^ (1 vvm). The pH was maintained at 7.0 by automatic addition of potassium hydroxide (3 M) or hydrochloric acid (2 M). The stirrer speed was fixed at 450 rpm, and a weir tube, coupled to a continuous pump, was fixed to the head-plate at a suitable height, such that 0.7 L working volume was maintained.

The chemostat seed-train began with a 10 mL LB culture inoculated from a single colony of strain CBC-3, CBC-9 or CBC-11 that was incubated for 12 h–16 h at 37°C with shaking at 250 rpm. MS medium (50 mL) for chemostat seed cultures was then inoculated to OD_600_ 0.1 from LB overnight cultures and incubated at 37°C with shaking at 250 rpm until OD_600_ 1.2 to OD_600_ 1.7. The bioreactor was then inoculated to an OD_600_ of 0.05–0.075 via an aspirator bottle that was connected to the bioreactor’s inoculation port with silicone tubing and pumping the inoculum into the bioreactor.

Following inoculation, the feed was turned on when the dissolved oxygen dropped below 80% of saturation, at a feed dilution rate of 0.1 h^−1^. The feed was started before the limiting nutrient had been consumed completely, thereby ensuring that cells did not reach stationary phase, where previous studies have suggested that genetic instability may be increased.^71^^,^^72^^,^^73^ This procedure was followed for all continuous culture experiments presented in this work. Dissolved oxygen never dropped below 30% DO. Samples were taken directly from the fermenter through a sterile sampling port.

#### Plasmid retention tests

Plasmid loss was determined by diluting culture samples and plating onto selective and non-selective plates. The loss of plasmid antibiotic resistance genes was assessed by serially diluting samples in 5 mM MgCl_2_, spreading aliquots on MS agar and incubating at 37°C for up to 40 h. Colonies (100) were then picked from plates with single colonies and transferred to both MS agar plates and antibiotic selective MS agar plates and incubated at 37°C for up to 40 h. The frequency of antibiotic resistance gene loss was calculated as a proportion of the colonies which were not viable on the antibiotic selective plate. The frequency of antibiotic resistance gene loss was calculated as a percentage of the number of colonies counted on the MS agar plates, versus those counted on the antibiotic selective plates.

#### Glucose, acetic acid, and citramalic acid quantification by HPLC

Citramalic acid, acetic acid, and residual D-glucose were determined via HPLC analysis using an Agilent 1200 Infinity series HPLC, equipped with both UV (210 nm) and refractive index indicators. Undiluted cultivation broth samples (1.5 mL) were prepared by centrifuging (4250 *× g*, 10 min) and filtering (0.2 μm pore filter). Samples were resolved using a Rezex ROA-Organic Acid H+ (8%), 30 × 4.6 mm column (Phenomenex), at 55°C with 0.01 N H_2_SO_4_ (0.5 mL min^−1^) as the mobile phase. Data analysis was performed with ChemStation software, using calibration curves prepared using standards of each compound (0.1–20 g L^−1^). HPLC analysis was performed in triplicate to provide technical repeats for each sample.

### Quantification and statistical analysis

Peak productivity for strains grown under glucose limited continuous culture is the mean peak productivity of three biological continuous culture repeats for strain CBC-5 (continuous cultures CC-1 to CC-3) and six biological continuous culture repeats of strain CBC-9 (continuous cultures CC-7 to CC-12), and the measure of dispersion is standard deviation.

Individual specific productivity measurements at given timepoints for each glucose limited and phosphate limited continuous fermentation is the mean of technical repeats, as indicated in the figure legend of Figure 4. The steady state specific productivity for phosphate limited continuous cultures of strain CBC-5 was calculated by taking the mean specific productivity across the steady state period for each of continuous cultures CC-4 to CC-6 (biological triplicates), and then taking the mean of these individual mean values. The measure of dispersion is the standard deviation across the three mean values, and the steady state period for each of CC-4, CC-5 and CC-6 is indicated in Table S1. The same method was used to calculate the average steady state productivity for phosphate limited continuous cultures of strain CBC-9 (continuous cultures CC-13 to CC15), taking the mean and standard deviations of the mean productivity across the steady state period for each of continuous cultures CC-13, CC-14 and CC-15, and the steady-state periods for each of these continuous cultures are indicated in Table S1.

The specific productivity for the phosphate limited continuous culture of strain CBC-11 (continuous culture CC-16) was the mean specific productivity measurement across the steady state period of the continuous culture. Since only one continuous culture was performed for this strain, no standard deviation across biological repeats was calculated.

The period of time reported for plasmid retention for strain CBC-5 under phosphate limitation is the mean number of hours across three continuous cultures (CC-4, CC-5 and CC-6; *n* = 3) for which 100% plasmid retention was observed, and the measure of dispersion is the standard deviation.

Cessation of productivity for strain CBC-9 grown under glucose limited continuous culture was considered to occur once specific productivity fell below 0.0025 g_CMA_ g_DCW_ h^−1^. The reported value for strain CBC-9 was calculated as the mean of all biological repeats, and the dispersion was calculated as the standard deviation (*n* = 6).
