# Supplementary material for: The generation game: Toward the generational genetic stability of continuous culture
Source: iScience. 2025 Jan 30;28(3):111787. doi: 10.1016/j.isci.2025.111787 (PMC11872498; doi:10.1016/j.isci.2025.111787)
Supplement: Document S1. Figures S1–S4 and Tables S1–S3 [file mmc1.pdf]

iScience, Volume *28*

Supplemental information

The generation game: Toward the generational genetic stability of continuous culture

Andrew Yiakoumetti, Charlotte Green, Mark Reynolds, John Ward, Gill Stephens, and Alex Conradie

Supplementary Information

Supplementary Figures:

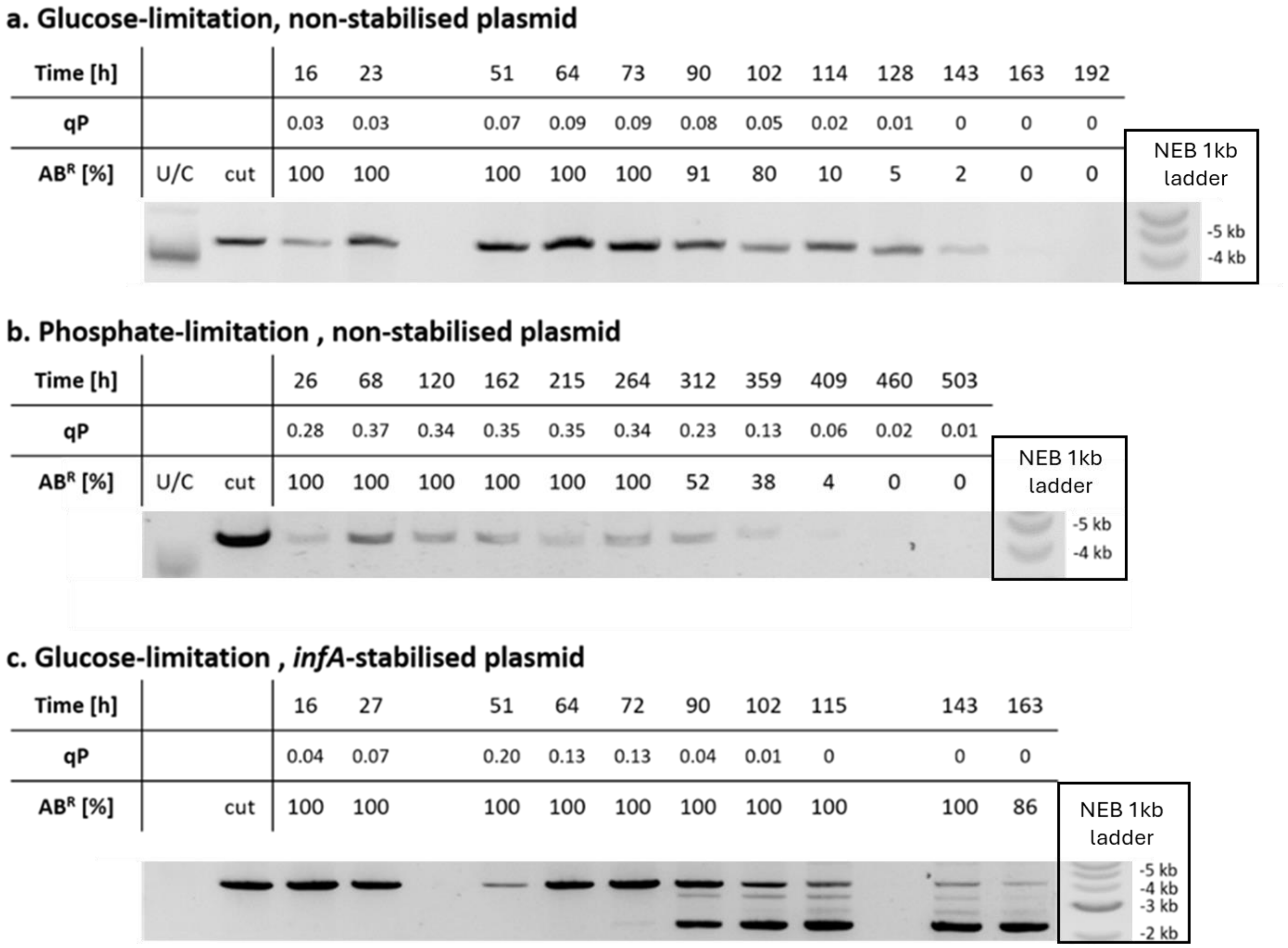

Fig. S1: Isolated plasmids, *Bam*HI digested; Related to Figure 4. qP is the specific productivity and AB^R is the measure of chloramphenicol sensitivity. U/C denotes uncut, where BamHI restiction enzyme was not included in reaction mixtures. (a) *Bam*HI digests of samples taken at different timepoints in Continuous Culture CC-03. (b) *Bam*HI digests of samples taken at different timepoints in Continuous Culture CC-06. (c) *Bam*HI digests of samples taken at different timepoints in Continuous Culture CC-09.

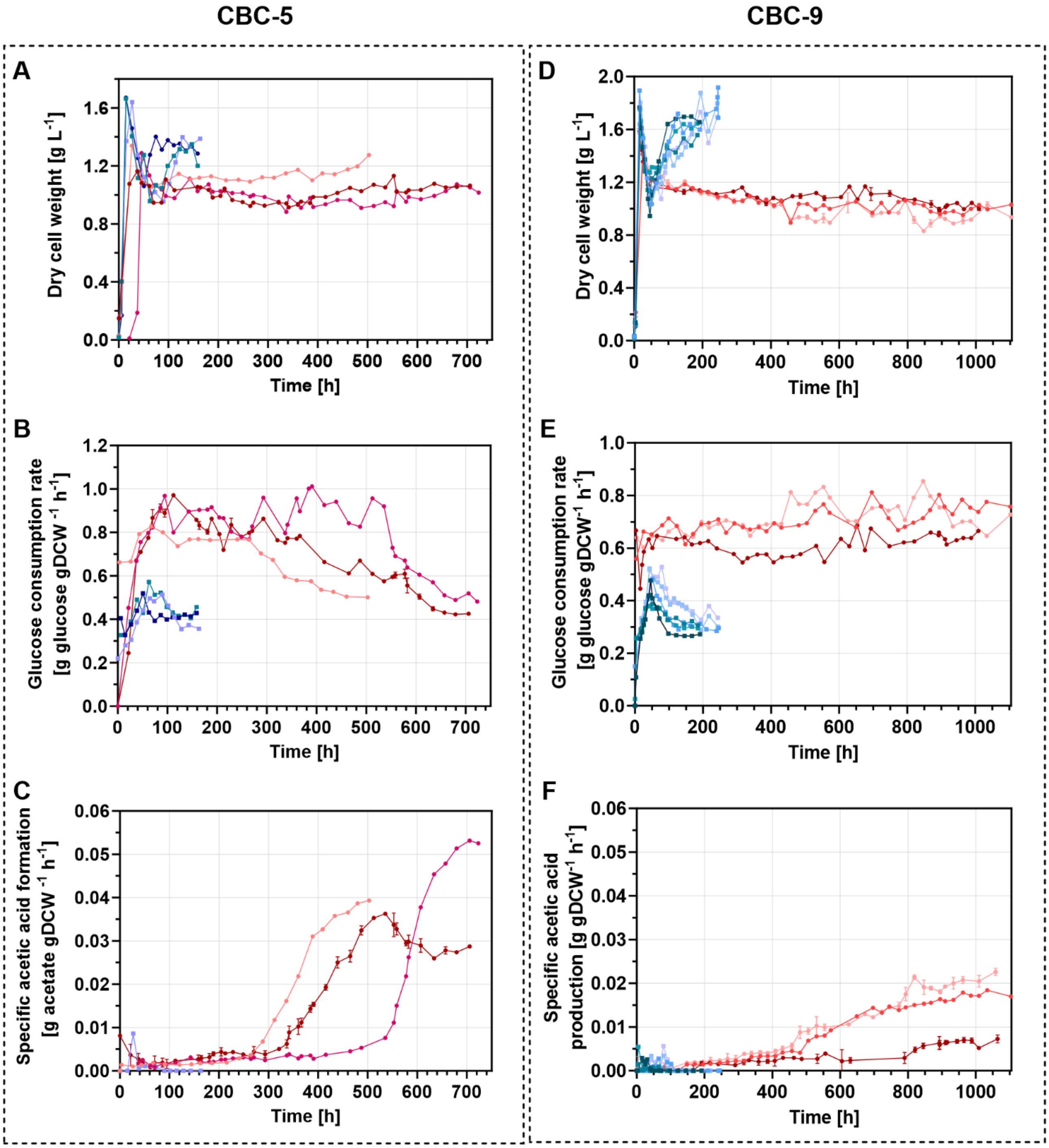

Fig. S2. Cell density, specific glucose consumption rate and specific acetate formation rate of the non-stabilised, plasmid-based CBC-5 strain and the *infA*-stabilised CBC-9 strain during glucose and phosphate limited continuous culture with a dilution rate of 0.1 h^{-1}; Related to Figure 4. Strains were grown in glucose (27.75 mM; blue shades) and phosphate (0.72 mM; red shades) limited continuous culture (minimum of 3 biological replicates). The feed was started when DO < 80 %, thus indicating cell growth. The figures show: (A, D) the cell density, (B, E) the specific glucose consumption rate and (C, F) specific acetate formation rate for CBC-5 and CBC-9 respectively. The mean and standard deviation of analytical technical replicates (N= 3) are displayed as error bars. The specific dry cell weight basis was calculated using the inlet dilution rate.

```
CC-07, COLONY 1: 945 NT DELETION, 10 NT CONTIGUOUS HOMOLOGY

UPSTREAM:   TGTCAGAGGTTTTCACCGTCATCACCGAAACGCGCGAGGCAGAAGGAGAT
                 *** * ****     **********      *       *
DOWNSTREAM: CTCAGGAGCTGTTCAAGAAAATCACCGAAAACGTTAACCTGCCGGTTAGC

PRODUCT:    TGTCAGAGGTTTTCACCGTCATCACCGAAAACGTTAACCTGCCGGTTAGC


CC-07, COLONY 2: 2555 NT DELETION, 7 NT CONTIGUOUS HOMOLOGY

UPSTREAM:   ACGGTTCCTGGCCTTTTGCTGGCCTTTTGCTCACATGTTCTTTCCTG
            *     *      ***  * *******        * **     *
DOWNSTREAM: AATAAGCACAAGTTTTATCCGGCCTTTATTCACATTCTTGCCCGCCT

PRODUCT:    ACGGTTCCTGGCCTTTTGCTGGCCTTTATTCACATTCTTGCCCGCCT


CC-07, COLONY 3: 1111 NT DELETION, 9 NT CONTIGUOUS HOMOLOGY

UPSTREAM:   GTTCTGACCCCGCAGAAGTCTCAGGAGCTGTTCAAGAAAATCACCGAAA
             ** ***         * * *********    ***  ****     **
DOWNSTREAM: TTTTTGAGTTATCGAGATTTTCAGGAGCTAAGGAAGCTAAAATGGAGAA

PRODUCT:    GTTCTGACCCCGCAGAAGTCTCAGGAGCTAAGGAAGCTAAAATGGAGAA


CC-08: 1854 NT DELETION, 5 NT CONTIGUOUS HOMOLOGY

UPSTREAM:   TCTTCGACACTACCCTGCGCGACGGCGAGCAGACCCCGGGTGTTA
              **    *   *  **   ***** **** *       * * **
DOWNSTREAM: AATTTCGTATGGCAATGAAAGACGGTGAGCTGGTGATATGGGATA

PRODUCT:    TCTTCGACACTACCCTGCGCGACGGTGAGCTGGTGATATGGGATA


CC-09: 1602 NT DELETION, 5 NT CONTIGUOUS HOMOLOGY

UPSTREAM:   GTCATCACCGAAACGCGCGAGGCAGAAGGAGATGGCGCCCAACAG
               * **        *  * ***** **      *  **** * *
DOWNSTREAM: TAAAACAGAATTTGCCTGGCGGCAGTAGCGCGGTGGTCCCACCTG

PRODUCT:    GTCATCACCGAAACGCGCGAGGCAGTAGCGCGGTGGTCCCACCTG
```

Fig. S3. Schematic demonstrating the regions of contiguous micro-homology (RED) between which deletions occurred, in the DNA regions upstream of the deleted region and downstream of the deleted region; Related to Figure 5. Broader imperfect homology between upstream and downstream regions are shown via asterix. DNA removed from upstream and downstream regions during the deletion event is shown in blue. After the deletion event, the DNA product retains DNA immediately upstream of the upstream region, and immediately downstream of the downstream region, as well as a single copy of a region of contiguous micro-homology.

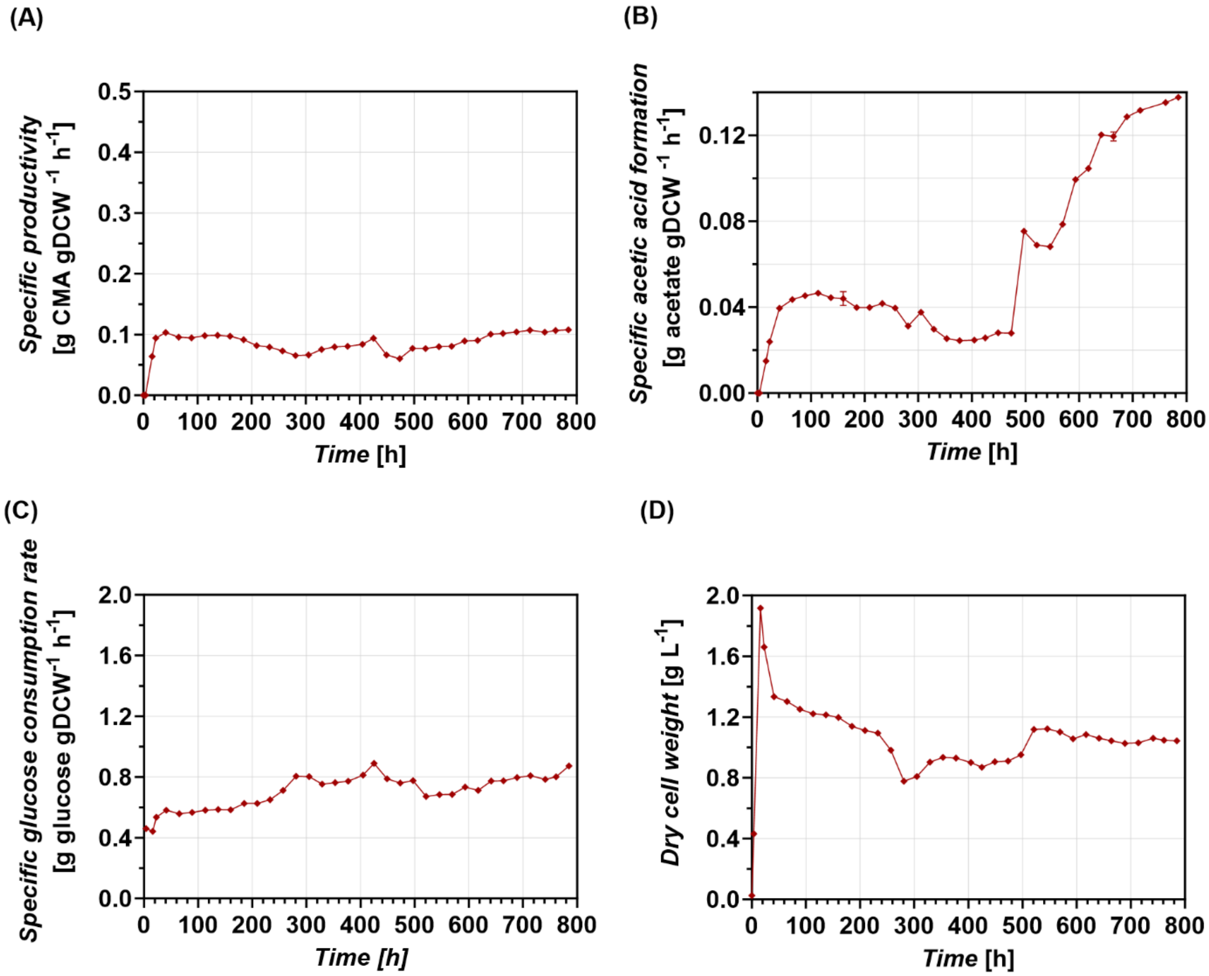

Fig. S4. Specific CMA productivity, specific acetate formation rate, specific glucose consumption rate and dry cell weight of the chromosomally integrated CBC-11 production strain during phosphate limited continuous culture; Related to Key Resources Table. (A) Specific citramalate productivity. (B) Specific acetate formation rate. (C) Specific glucose consumption rate. (D) Dry cell weight. The feed (inlet D = 0.1 h^{-1}) was started when DO < 80 %, thus indicating cell growth. One biological replicate. The specific dry cell weight basis was calculated using the inlet dilution rate. During the first 71 generations, acetate was produced at an average of 0.04 $g_{ACE}\cdot g_{DCW}^{-1}\cdot h^{-1}$. However, acetogenesis increased sharply thereafter to reach a final value of 0.14 $g_{ACE}\cdot g_{DCW}^{-1}\cdot h^{-1}$.

Supplementary Tables

Table S1: Continuous cultures performed in this study under glucose and phosphate limitation at an inlet dilution rate of 0.1 h^{-1} and 37°C for strains CBC-5, CBC-9 and CBC-11; Related to Figure 4. Only strains CBC-5 and CBC-9 were grown under both glucose limitation and phosphate limitation. Strain CBC-11 was only grown under phosphate limitation. The measure of dispersion for longevity is standard deviation. *The ~120 h of reported productive continuous culture for strain CBC-5 under glucose limitation is relevant for two of the three biological repeats. Another biological repeat was productive for only 75 h.

Continuous Culture ID	Strain	Limitation	Measure of longevity (Type and mean length in h)	Steady-state period	Steady state productivity across biological repeats ($g_{CMA} \cdot g_{DCW}^{-1} \cdot h^{-1}$)
CC-01	CBC-5	Glucose	Cessation of productivity after ~120 *	No steady state	No steady state
CC-02				No steady state	
CC-03				No steady state	
CC-04		Phosphate	Plasmid loss observed after 324 ± 80.4	45.8 h – 360 h	0.35 ± 0.02
CC-05				61.3 h – 384 h	
CC-06				43.4 h – 264 h	
CC-07	CBC-9	Glucose	Cessation of productivity after 153 ± 25.4	No steady state	No steady state
CC-08				No steady state	
CC-09				No steady state	
CC-10				No steady state	
CC-11				No steady state	
CC-12				No steady state	
CC-13		Phosphate	Culture remained productive for >1000	25.0 h to end of culture	0.32 ± 0.01
CC-14				49.4 h to end of culture	
CC-15				25.8 h to end of culture	
CC-16	CBC-11		Culture remained productive for >785	23 h to end of culture	0.088

Table S2: Sequence analysis summary for glucose limited continuous cultures of strain CBC-9; Related to Figure 5.

**Continuous culture**	**Observed plasmid structural mutation**
CC-07 (Three colonies sequenced. Different mutations observed in each)	Colony 1's plasmid sequence revealed the complete deletion of the J23104 promoter and partial truncation of *cimA3.7* (945 nt deletion).
	Colony 2's plasmid sequence showed complete deletion of the *cimA3.7* expression cassette, deletion of the CAT promoter and partial deletion of *cat* (2555 nt deletion).
	Colony 3's plasmid sequence exposed partial truncation of *cimA3.7* alongside complete deletion of the CAT promoter and partial deletion of *cat* (1111 nt deletion).
CC-08	Partial truncation of *cimA3.7* alongside complete deletion of the CAT promoter and partial deletion of *cat* (1854 nt deletion). Chloramphenicol sensitivity observed at the point of sequencing.
CC-09	Complete deletion of the *cimA3.7* expression cassette (1602 nt deletion). Chloramphenicol sensitivity arises after the point of sequencing.
CC-10	A single nucleotide deletion resulted in a frameshift mutation in *cimA3.7*. Chloramphenicol sensitivity arises after the point of sequencing.
CC-11	A single nucleotide substitution resulted in a missense mutation in *cimA3.7*. Same mutation observed in both plasmid isolates sequenced.
CC-12	No plasmid sequencing, but culture becomes chloramphenicol sensitive, indicating genetic disruption of *cat* expression cassette

Table S3: Oligonucleotides (oligos) used in this study and promoter fragments constructed in this study by annealed phosphorylated oligonucleotide pairs; Related to Key Resources Table.

**Oligo ID**	**Sequence (5' to 3')**
OLIGO-1	GCGATGGCTAGCAGGAGGAATTCAC
OLIGO-2	AGTTGTGGATCCTTGGTAACGAATCAGACAATTGAC
OLIGO-3	GATCCTAATACGACTCACTATAGCTCGAGACCCGTTTTTTTGGG
OLIGO-4	CTAGCCCAAAAAAACGGGTCTCGAGCTATAGTGAGTCGTATTAG
OLIGO-5	GATCCTTGACAGCTAGCTCAGTCCTAGGTATAATGCTAGCC
OLIGO-6	TCGAGGCTAGCATTATACCTAGGACTGAGCTAGCTGTCAAG
OLIGO-7	GATCCTTGACAGCTAGCTCAGTCCTAGGTATTGTGCTAGCC
OLIGO-8	TCGAGGCTAGCACAATACCTAGGACTGAGCTAGCTGTCAAG
OLIGO-9	GATCCCTGACAGCTAGCTCAGTCCTAGGTATAATGCTAGCC
OLIGO-10	TCGAGGCTAGCATTATACCTAGGACTGAGCTAGCTGTCAGG
OLIGO-11	GATCCTTTACGGCTAGCTCAGTCCTAGGTACTATGCTAGCC
OLIGO-12	TCGAGGCTAGCATAGTACCTAGGACTGAGCTAGCCGTAAAG
OLIGO-13	GATCCTTTATGGCTAGCTCAGTCCTAGGTACAATGCTAGCC
OLIGO-14	TCGAGGCTAGCATTGTACCTAGGACTGAGCTAGCCATAAAG
OLIGO-15	GATCCCTGATAGCTAGCTCAGTCCTAGGGATTATGCTAGCC
OLIGO-16	TCGAGGCTAGCATAATCCCTAGGACTGAGCTAGCTATCAGG
OLIGO-17	CATATGCTGTCAGACCAAGTTTACTCATATATACTTTAGATT
OLIGO-18	AGAGTTTGTAGAAACGCAAAAAGGCCATCCG
OLIGO-19	TAATCTAGACAGAGGATTAGATGGCCAAAGAAGACAATATTGAAATGC
OLIGO-20	GTAAACTTGGTCTGACAGCATATGTTAGCGACTACGGAAGACAATG
OLIGO-21	CCTTTTTGCGTTTCTACAAACTCTTGATCGGCACGTAAGAGGTTC
OLIGO-22	CATCTAATCCTCTGTCTAGATTACGCCCCGCCCTG
OLIGO-23	TATCTTGCCGGTTCAAATTACGGTAGTGATACCCCAGAGGATTAGTTGCC AGCTGGGGCGCCCTCTG
OLIGO-24	ACCTTTTACTCGTTCTTTCTCTTCGCCCATCAGGCGGTAAAACAATCAGA AGAACTCGTCAAGAAG
OLIGO-25	CTGTCAAACATGAGAATTAATTCCGGGGATCCG
OLIGO-26	ATTGTGTAGGCTGGAGCTGCTTCG
OLIGO-27	GGTTTGGTCATTGTTAATGAGCACTGACAGCTAGCTCAGTCC
OLIGO-28	CTTCGAAGCAGCTCCAGCCTACACAATTTGTCCTACTCAGGAGAGC
OLIGO-29	GGTTTGGTCATTGTTAATGAGCATTGACAGCTAGCTCAGTCC
OLIGO-30	GAGAATACGGTAGTAAGTGAGATATGGGGTTTGGTCATTGTTAATGAGC A
OLIGO-31	CACCAATATAAACCAAGGAAGAATCCAGTCGTTGGCGGTCATGATTGTC ACTGTCAAACATGAGAATTAATTCCG

Promoter	Phosphorylated Oligonucleotide 1	Phosphorylated Oligonucleotide 2
T7	OLIGO-3	OLIGO-4
J23119	OLIGO-5	OLIGO-6
J23104	OLIGO-7	OLIGO-8
J23108	OLIGO-9	OLIGO-10
J23105	OLIGO-11	OLIGO-12
J23114	OLIGO-13	OLIGO-14
J23112	OLIGO-15	OLIGO-16
